# Fabrication of MnCuNiFe–CuAlNiFeMn Gradient Alloy by Laser Engineering Net Shaping System

**DOI:** 10.3390/ma15062336

**Published:** 2022-03-21

**Authors:** Kuo Yan, Zaiwen Lin, Meng Chen, Yuren Wang, Jun Wang, Heng Jiang

**Affiliations:** 1 College of Material Science and Chemical Engineering, Harbin Engineering University, Harbin 150001, China; yankuo1234@126.com (K.Y.); zhqw1888@sohu.com (J.W.); 2 Key Laboratory of Microgravity, Institute of Mechanics, Chinese Academy of Sciences, Beijing 100190, China; chenmeng@imech.ac.cn (M.C.); yurenwang@imech.ac.cn (Y.W.); 3 University of Chinese Academy of Sciences, Beijing 100049, China

**Keywords:** propeller noise reduction, gradient alloys, damping alloys, additive manufacturing, 3D printing

## Abstract

Marine noise pollution generated by propellers is of wide concern. Traditional propeller materials (nickel–aluminum bronze (NAB) alloys) can no longer meet the requirements for reducing shaft vibration. However, the Mn–Cu alloy developed to solve the problem of propeller vibration is affected by seawater corrosion, which greatly limits the application of the alloy in the field of marine materials. In this study, the M2052–NAB gradient alloy was developed for the first time using LENS technology to improve the corrosion resistance while retaining the damping properties of the M2052 alloy. We hope this alloy can provide a material research basis for the development of low-noise propellers. This study shows that, after solution-aging of M2052 alloy as the matrix, the martensitic transformation temperature increased to approach the antiferromagnetic transformation temperature, which promoted twinning and martensitic transformation. The aging process also eliminated dendrite segregation, promoted the equiaxed γ-MnCu phase, and increased the crystal size to reduce the number of dislocations, resulting in obvious modulus softening of the alloy. NAB after deposition had higher hardness and good corrosion resistance than the as-cast alloy, which offers good corrosion protection for the M2052 alloy. This research provides new material options for the field of shipbuilding.

## 1. Introduction

The damage of noise pollution to the marine environment has received widespread attention in recent years [1]. The main noise sources of a ship in motion are hydrodynamic, mechanical, and propeller noise; propeller noise is mainly due to cavitation and vibration [2,3]. Relevant practitioners in the shipbuilding industry have long been concerned by the noise of propellers, and related research on reducing propeller noise is ongoing. In terms of cavitation noise, researchers have conducted a numerical analysis of noise models [2,4,5], blade structure optimization [3,6,7], and surface treatment [8]. The current solution to vibration and noise between the propeller and the main shaft is to install an additional vibration isolation system [9,10], but this occupies the interior space of the ship, complicates the transmission system, and cannot resist vibration. It is desirable to solve the root cause of the problem.

Mn–Cu alloy was once regarded as an ideal material for propeller damping, shock absorption, and noise reduction due to its excellent damping and mechanical properties [11,12,13]; however, due to its severe shrinkage porosity and poor stress corrosion and corrosion fatigue resistance, Mn–Cu alloy still faces challenges for application in the field of propeller manufacturing [12,14]. Current marine propeller materials are still mainly nickel–aluminum bronze (NAB) and composite materials [15,16,17,18,19,20,21]; Mn–Cu damping alloys have not been widely used. In order to solve the above problems, researchers used traditional sintering [22] or cumulative rolling [23] methods to prepare gradient composites. Although the alloy properties can be improved, these traditional processing methods are not suitable for propellers, which are parts with complex geometries.

In recent years, additive manufacturing (AM) technology has attracted extensive interest from researchers as a new processing scheme. AM, also known as 3D printing technology, is a rapid prototyping method based on digital model files to prepare materials with a certain three-dimensional (3D) structure through layer-by-layer printing [24,25]. Three-dimensional printing uses less material than traditional manufacturing techniques, shortens the production cycle, and has lower design costs and operational difficulties [26]. This technology has advantages over traditional processing in the rapid manufacture of complex structural parts and the development of gradient composite materials [27,28]. In the field of propeller manufacturing, 3D printing technology has a certain research base. Khaleed et al. developed a propeller manufacturing method for autonomous underwater vehicles by fused deposition modeling [29]. He et al. used wire arc AM to obtain metal propellers that gave better mechanical properties than those of cast propellers [30]. Liu et al. used laser melting deposition to achieve AM preparation of ^83^Cu_9_Al_4_Ni_3.5_Fe_0.5_Mn alloy for marine propeller materials [16], the mechanical properties of which were also better than those of traditional casting materials.

At present, the use of 3D printing technology to manufacture gradient alloy propellers has not been reported. This study will be the first to use laser engineering net shaping technology to develop propeller materials with both noise reduction and corrosion resistance. Laser engineering net shaping (LENS) is a type of AM in which parts are processed layer by layer through coaxial powder feeding. The powder ratio can be freely adjusted during processing, which has obvious advantages in the preparation of vertical gradient materials [31,32,33]. In terms of materials, we chose M2052 alloy, widely used in the field of vibration and noise reduction, and developed M2052–NAB gradient alloy based on NAB alloy, the mainstream material in the field of marine propellers [34,35,36]. The M2052 alloy was used as the material of the propeller hub to improve vibration reduction of the shaft. As part of the blade, the NAB alloy not only protects the M2052 alloy to ensure corrosion resistance in a marine environment, but its excellent machinability can achieve the necessary subsequent processing and surface treatment of propeller blades [15,37,38]. At the same time, because the 3D printing process has good designability, the gradient material processing technology developed in this study can provide a research basis for the processing technology of gradient alloy propellers.

## 2. Materials and Methods

### 2.1. Materials and Preparation

**Figure 1:**
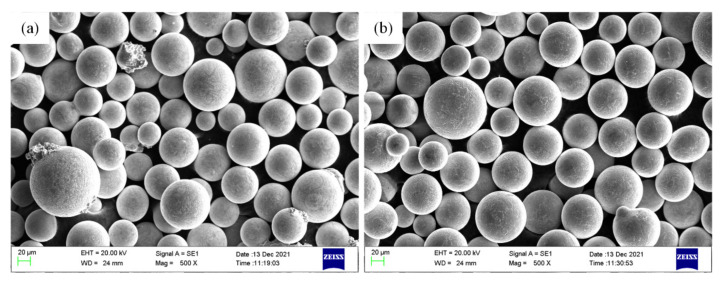
Particle morphologies of (**a**) M2052 and (**b**) NAB.

The test materials were M2052 alloy and NAB alloy spherical powders (Shaanxi Yingbo Metal Technology Co., Ltd., Weinan, China) prepared by a plasma rotating electrode process. The powder particle size was 75–150 μm. The appearances of the two powders are shown in Figure 1. The spherical forms were generally well preserved, and only a small amount of powder was damaged. Table 1 shows the compositions of the powders determined by energy-dispersive spectrometry (EDS) and the change in alloy chemistry after LENS processing. The powder LENS system used an infrared fiber laser with a power of 400 W, a spot diameter of 500 μm, and a laser wavelength of 1070 nm. In order to ensure printing quality, the oxygen content was held below 60 ppm during the printing process.

The additive processing parameters are shown in Table 2. Stainless steel 316L alloy with a size of 100 mm × 100 mm × 10 mm was used as the printing substrate. The M2052 alloy was printed on the substrate first. The samples were solution-treated at 1153 K for 2 h and then aged at 708 K for 6 h after water quenching. The surface of the aged sample was polished and cleaned to remove the oxide layer, then it was replaced in the printing position and the NAB alloy deposited. According to Table 1, the overall chemical composition of the alloy changed little after the powder was deposited and formed by the LENS process. The proportion of Mn in both alloys was reduced, which may be caused by the metal vapor generated during the printing process due to the volatile nature of Mn [26]. In order to reduce the influence of oxygen on the material, the inside of the forming chamber is protected by argon gas. During the forming process, the oxygen content in the forming chamber was controlled at <30 PPM, and no obvious O residue was found in the alloy after forming.

### 2.2. Material Characterization Method

A Q800 dynamical mechanical analyzer (DMA; TA Instruments, Newcastle, PA, USA) was used to compare and analyze the M2052 alloy directly deposited and after aging. Temperature-testing of the samples at 0.1 Hz, 1 Hz, and 10 Hz was carried out using the three-point bending mode, with a temperature sweep range of 143–523 K, heating rate of 5 K/min, and amplitude of 5 × 10^−5^. tan δ at different frequencies and storage modulus (*E*) was measured as a function of temperature (*T*).

The samples of the M2052 alloy as-deposited directly by LENS were cut and prepared after solution-aging. After mechanical polishing, electrolytic polishing was performed with a mixed solution of ethanol, phosphoric acid, and glycerol (2:1:1). The M2052–NAB alloy was mechanically polished and etched with 3% FeCl_3_ ethanol solution. The alloy microstructures of the metallographic samples were observed with a Leica DM 4M optical microscope (Leica, Wetzlar, Germany). The crystal structure was determined by X-ray diffraction (XRD; D/max-rA, Rigaku, Japan) using a 2θ scanning range of 20–100° and a step size of 0.02°.

The microstructure at the interface of the M2052–NAB gradient alloy was analyzed by line-drawing analysis of the elemental composition on both sides of the interface by scanning electron microscopy (SEM; LEO-1450, LEO Co., Oberkochen, Germany) with EDS function. The microhardness changes on both sides of the interface were measured using an MH-6 microhardness tester (Hengyi, Shanghai, China).

The NAB alloy was tested for corrosion using a CS310 electrochemical workstation (KOST Instruments, Wuhan, China). The electrochemical properties of the samples in 3.5% NaCl solution were measured at room temperature. The sample was the working electrode, a saturated calomel electrode was the reference electrode, and a platinum electrode was the auxiliary electrode. The scanning range of the potentiodynamic polarization curve was −0.5–1 V, and the scanning speed was 0.333 mV/s. The self-corrosion potential (E_corr_) and current density (I_corr_) were obtained by Tafel fitting.

## 3. Results and Discussion

**Figure 2:**
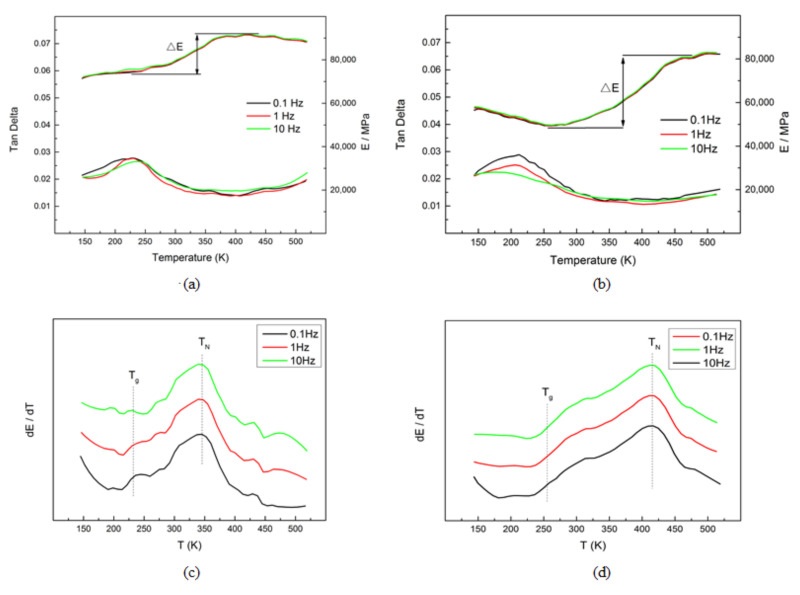
Relationship between tan δ, Young’s modulus, and temperature of the M2052 alloy (**a**) as-deposited and (**b**) after aging. Relationship between d*E*/d*T* and temperature of M2052 alloy (**c**) as-deposited and (**d**) after aging.

Figure 2 shows dynamic mechanical spectra of the M2052 alloy in the as-deposited state and after aging. Both alloy samples produced a slightly raised twinning peak near 220 K, the position of which gradually shifted to a higher temperature with the increase in frequency. This indicated the relaxation effect of coincidence frequency on twinning [35]. M2052 alloy is an antiferromagnetic material, so it is distorted due to lattice instability when the antiferromagnetic transition occurs and the symmetry changes, resulting in modulus softening [13,34]. After aging, softening of the modulus was more obvious: Δ*E* increased from 17,000 MPa in the as-deposited state (Figure 2a) to 33,000 MPa (Figure 2b), which indicated that the martensitic transformation temperature *T*_M_ approached the antiferromagnetic transformation temperature *T*_N_ (d*E*/d*T*). Lattice distortion and martensitic transformation of the M2052 alloy are coupled when antiferromagnetic transformation occurs, so Δ*E* increases. Furthermore, AM is characterized by rapid cooling, so the grain size of the as-deposited sample was smaller than that of the aged alloy sample. The as-deposited state had more dislocations, which inhibit lattice distortion, and softening of the modulus was less obvious. The aged alloy exhibited amplitude-modulated decomposition and *T*_N_ in the Mn-rich region was enhanced (Figure 2d), thereby promoting antiferromagnetic transformation and twinning, which are accompanied by martensitic transformation.

**Figure 3:**
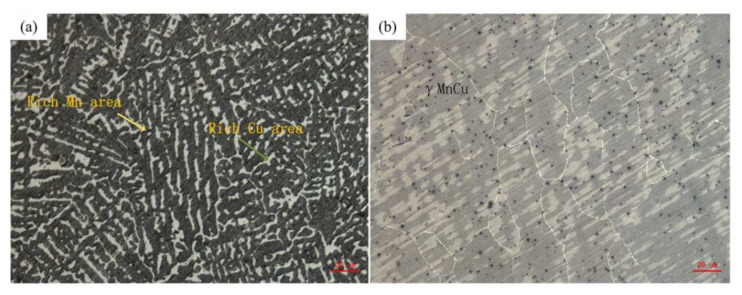
Microstructures of M2052 (**a**) as-deposited and (**b**) after aging.

The microstructure of the as-deposited M2052 alloy was the same as that of the as-cast state, and obvious dendrite segregation occurred. The dark and bright regions depict the Mn- and Cu-rich regions, respectively (Figure 3a) [11]. There was no obvious dendrite growth or orientation. Due to the high melting point of Mn, Mn first precipitates out to form an Mn-rich region of the dendritic structure during solidification. The Mn content is about 80%, and the Cu content is about 10%. Cu with a lower melting point is precipitated between dendrites to form a Cu-rich region with an Mn content of about 60% and a Cu content of about 35% [39,40]. After aging, the dendrites partially melted to form a uniform γ-MnCu equiaxed crystal system, the grain size increased significantly, and the dislocation density was reduced (Figure 3b). There were obvious pore defects on the surface of the alloy samples. Combined with the elemental composition analysis (Table 1), this is attributed to pores generated by the volatilization of Mn vapor. Pore formation is the main problem faced by the current 3D printing process: a large number of defects affect the mechanical properties of a material, and its performance is adversely affected [26].

**Figure 4:**
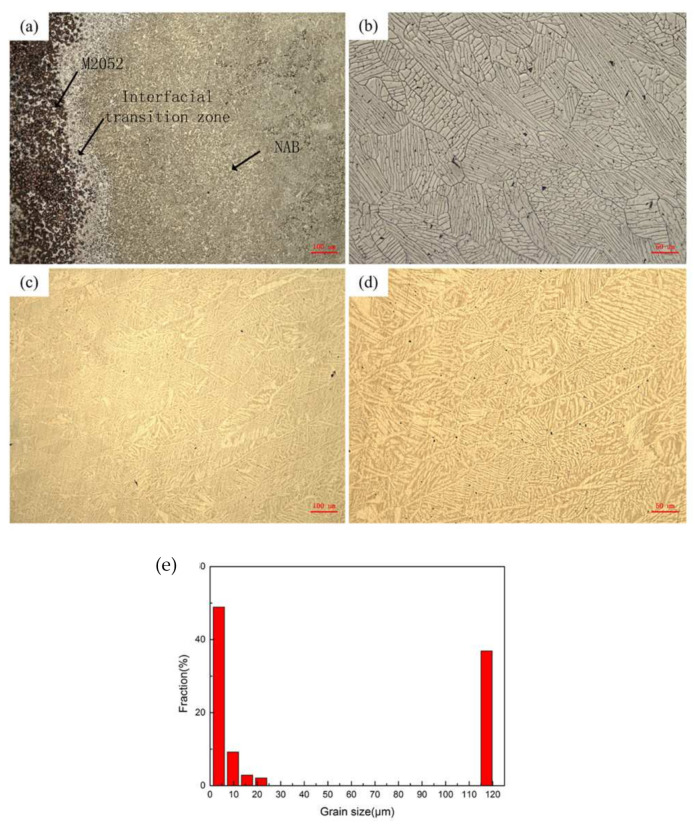
Microstructures of (**a**) M2052–NAB gradient alloy, (**b**) transition zone, (**c**) NAB alloy, and (**d**) NAB alloy surface pore defects, (**e**) Grain size distribution of M2052–NAB alloy.

Deposition of NAB alloy produced a clear transition zone after deposition of the M2052 alloy (Figure 4a), of approximately 100 μm in width. The transition layer gradually dissolved on both sides, which is quite different from the obvious interface delamination observed in welding and hot-pressing processes. The transition zone comprised mainly disordered columnar crystals (Figure 4b). The main structure of NAB alloy is α-phase, which is FCC lattice solid solution with Cu as the main body. β’ is a martensitic structure formed by the transformation of high-temperature disordered β-phase (BCC) and κ-phase (Fe, Ni, Al eutectoid) [38]. Due to the rapid cooling of the 3D printing, the alpha phase is precipitated in the form of needles. The α-phase grew inward along the boundary to produce a feather-like structure similar to that of Widmanstatten. Some relatively coarse martensite β’ was also present. Irregular pore defects caused by melt pool collapse during printing were found in the transition zone, and NAB alloy (Figure 4c); circular pores distributed on the substrate surface were visible at higher magnification (Figure 4d). The grain size distribution of M2052–NAB alloy shows obvious polarization, with the grain size of 3.73 μm accounting for 48.9% and the grain size of 117.4 μm accounting for 36.87% (Figure 4e). Combined with the analysis in Figure 3b, the larger grain size is for the aged M2052 alloy. The smaller grain size is the as-deposited NAB alloy. Since 3D printing is a rapid cooling process, NAB has a smaller grain size during deposition.

**Figure 5:**
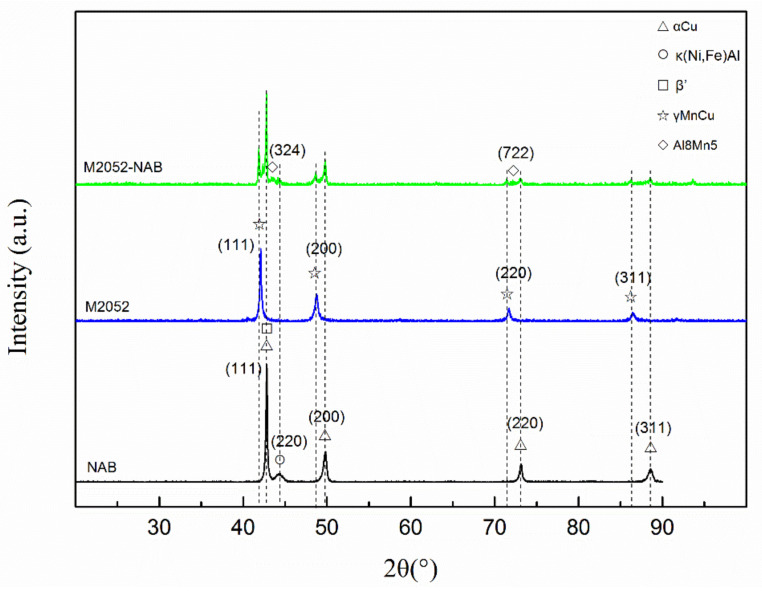
X-ray diffraction patterns of NAB, M2052, and M2052–NAB gradient alloy.

As shown by the XRD pattern (Figure 5), the crystal structure of the as-deposited NAB alloy is dominated by α phase, the Miller indices of characteristic peaks are (111), (200), (220), (311), and the diffraction peak of K phase (220) appears at 44°. The β’ phase and the α phase are difficult to distinguish. According to the Miller index, the M2052 alloy mainly exhibited diffraction peaks of the γ-MnCu phase [40]: no diffraction patterns of other crystals were found. In addition to exhibiting the same crystal structure as the M2052 and NAB alloys, the XRD patterns of M2052–NAB gradient alloys also had Al–Mn diffraction peaks; Miller exponent is (324), (722). This phase only occurred in the transition region. This indicates that during the deposition of NAB on the M0252 matrix, the newly emerged Al interacts with Mn in the matrix to form an Al–Mn phase.

**Figure 6:**
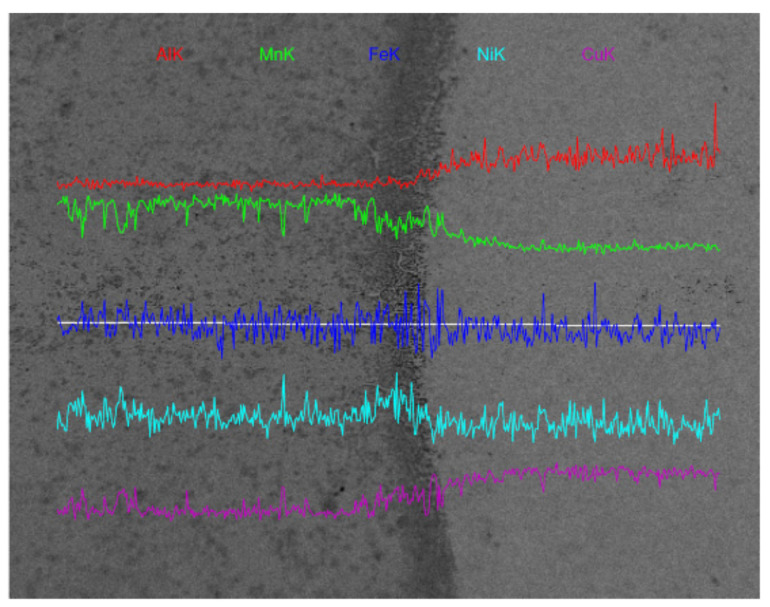
Changes in element concentrations from M2052 to NAB across the interface, as measured by energy-dispersive spectroscopy.

Figure 6 shows the EDS analysis of the M2052–NAB alloy. Fluctuation of the energy spectrum curve indicates that element segregation occurred in the material. The dark region in the figure is the transition zone between the M2052 alloy on the left and the NAB alloy on the right. The Mn, Cu, and Al contents changed more obviously on both sides of the interface: that of Mn decreased on the right side of the interface, the Al and Cu contents increased, while those of Fe and Ni did not change significantly. The changes in Mn, Cu, and Al contents in the transition zone are relatively smooth, which indicates that the two alloys had good mutual fusion and the fully miscible transition zone provided good interfacial properties for their combination.

**Figure 7:**
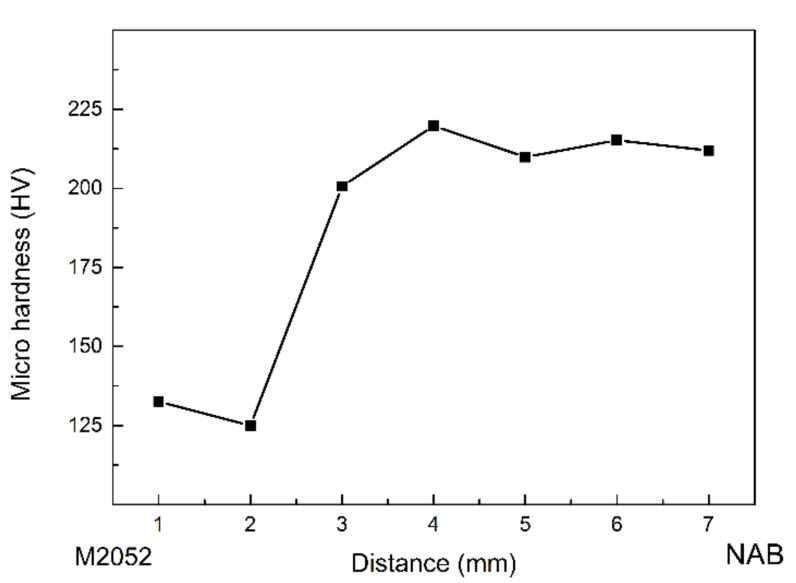
Microhardness of graded material from M2052 to NAB.

Figure 7 shows the microhardness change for the M2052–NAB gradient alloy, indicating a significant increase during the transition from M2052 to NAB. The microhardness of NAB reached 215 HV, which is higher than that of as-cast NAB but lower than that of the product after friction stir welding [37]. This is because deposition by LENS results in rapid cooling, which gives rise to a small grain size with no obvious orientation, and a large number of dislocations are generated, which improves the hardness. The part with reduced hardness is the M2052 alloy. After aging, the grain size of the M2052 alloy increases, the dislocation density decreases, and the hardness of the alloy decreases significantly.

The main reason for the corrosion of NAB in 3.5% NaCl solution is the oxidation of Cu at the anode to form an oxide layer [17,18]. The chemical reaction equation is:

$${\mathrm{Cu}+2\mathrm{Cl}}^{-}\;\to {\;\mathrm{CuCl}}_{2}^{-}{+e}^{-};$$

$${2\mathrm{CuCl}}_{2\;}^{-}{+H}_{2}O\;\to {\;\mathrm{Cu}}_{2}{O+2H}^{+}{+4\mathrm{Cl}}^{-};$$

$${\mathrm{Cu}}_{2}{O+\mathrm{Cl}}^{-}{+2H}_{2}O\;\to {\;\mathrm{Cu}}_{2}{\left(\mathrm{OH}\right)}_{3}{\mathrm{Cl}+H}^{+}{+2e}^{-};$$

Al in NAB alloy can provide better corrosion resistance, and Al will form a dense layer of Al_2_O_3_ on the surface of the substrate. Al_2_O_3_ is denser than Cu_2_O and has no electrical conductivity. It can effectively isolate the NAB matrix from the corrosive medium 3.5% NaCl solution and block its charge exchange, thereby providing better protection [41,42]. The reaction equation is:

$${\mathrm{Al}+4\mathrm{Cl}}^{-}\;\to {\;\mathrm{AlCl}}_{4\;}^{-}{+3e}^{-};$$

$${2\mathrm{AlCl}}_{4}^{-}{+3H}_{2}O\;\to {\;\mathrm{Al}}_{2}O_{3}{+6H}^{+}{+8\mathrm{Cl}}^{-}.$$

**Figure 8:**
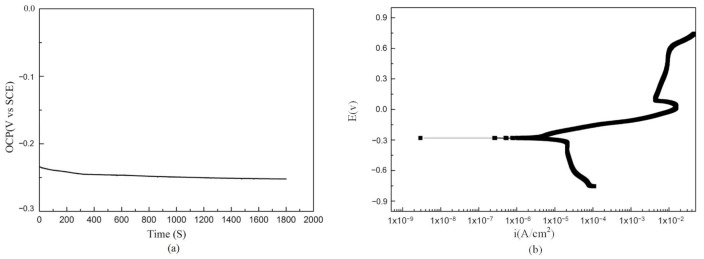
(**a**) Stable open circuit potential of NAB deposited by LENS in 3.5% NaCl. (**b**) Dynamic polarization curve of electrochemical corrosion of NAB.

NAB was electrochemically corroded in 3.5% NaCl, and a stable open circuit potential was obtained at −0.25 V (Figure 8a). Analysis of the polarization curve showed that the corrosion potential of the sample was −280 mV and the self-corrosion current density was 7.24 × 10^−6^ A/cm^2^. The corrosion potential of NAB deposited by LENS does not differ much from that of Selective Laser Melting and as-cast alloys, but the self-corrosion current density is significantly reduced. This indicates that the NAB alloy deposited by LENS has good corrosion resistance in 3.5% NaCl and can provide corrosion protection for the M2052 substrate [38].

## 4. Conclusions

An M2052–NAB gradient alloy was developed using the LENS process. Dynamic mechanical analysis of the as-deposited M2052 alloy and after aging was carried out. The microstructural morphology, composition analysis, and microhardness of each region of the gradient material were observed. The corrosion resistance of NAB alloy in 3.5% NaCl after deposition was analyzed by electrochemical corrosion testing. The main results include the following:

1. The microstructure of the as-deposited M2052 alloy was the same as that of the as-cast alloy, with obvious Mn-rich regions and dendrite segregation in the Cu-rich regions. After aging, the dendrite structure in the Mn-rich region disappeared, and the alloy microstructure was equiaxed γ-MnCu. The grain size increased, dislocation density decreased, and apparent modulus softening occurred. These results show that the aged alloy promoted the formation of twins and martensite;
2. A transition zone with a width of about 100 μm was formed at the interface of the M2052–NAB gradient alloy, and the alloy had no obvious grain orientation. In XRD analysis, an additional Al–Mn phase appeared in the gradient alloy, indicating that Al in the NAB alloy interacted with Mn in the M2052 matrix to form a new phase in the transition zone. Microhardness testing showed that the hardness of NAB alloy in the as-deposited state was higher than in the as-cast state but lower than that of the alloy after friction stir welding;
3. The NAB alloy deposited by LENS showed good corrosion resistance in 3.5% NaCl. The corrosion potential of −0.28 V was comparable with that of the as-cast and SLM alloys. The self-corrosion current density was 7.24 × 10^−6^ A/cm^2^, which is lower than that of the as-cast alloy and SLM deposited alloys;
4. The deposited alloy had tiny pores and irregular pore defects caused by molten pool collapse, which affected the material properties. The printing parameters of the alloy have not yet been analyzed and optimized, so there is still potential for improvement in the performance of the gradient alloy;
5. Compared with existing studies, the M2052–NAB alloy interface possesses a good metallurgical bond [32], and the corrosion resistance in the 3.5% NaCl solution is higher than that of the as-cast and SLM deposited alloys.

This study develops an M2052–NAB gradient alloy that retains the damping properties of the alloy while also exhibiting corrosion resistance. The LENS process preparation technology makes the alloy have better designability and can provide new material options for valves, propellers, and subsea pipelines. 

We conducted a preliminary exploration of the fabrication process of M2052–NAB gradient alloy. The mechanical properties of the alloy and the optimization of processing parameters will be further studied in the follow-up work. However, the research on gradient alloys is still insufficient. The current process parameters are not the optimal solution, and the biofouling, cavitation corrosion, electrochemical corrosion, and other properties of the alloy in the marine environment still need to be studied and optimized. We will delve into these issues in future work.

## Figures and Tables

**Table 1:** Chemical compositions (mass%) of M2052–NAB powder and M2052–NAB alloy prepared by LENS.

	Mn	Cu	Ni	Fe	Al	O
NAB powder	0.6	79.0	4.3	3.6	12.5	0.0
LENS–NAB alloy	0.4	81.4	4.3	3.5	10.4	0.0
M2052 powder	69.6	21.4	6.0	2.9	-	0.0
LENS–M2052 alloy	68.8	21.5	5.8	3.8	-	0.0

**Table 2:** Processing parameters of M2052–NAB sample.

Parameters	Value
Laser Power (W)	300
Scanning Speed (cm/min)	50
Powder Feeder (r/min)	4
Layer Thickness (μm)	254
Powder Carrier Gas (MPa)	0.04
Shielding Gas (MPa)	0.19
Atmospheric oxygen content (PPM)	<30

## Data Availability

The data presented in this study are available.
